# Cloud-based biomedical data storage and analysis for genomic research: Landscape analysis of data governance in emerging NIH-supported platforms

**DOI:** 10.1016/j.xhgg.2023.100196

**Published:** 2023-04-12

**Authors:** Jacklyn M. Dahlquist, Sarah C. Nelson, Stephanie M. Fullerton

**Affiliations:** 1 Department of Bioethics and Humanities, University of Washington School of Medicine, Seattle, WA 98195, USA; 2 Department of Biostatistics, University of Washington, Seattle, WA 98195, USA

**Keywords:** data sharing, cloud platforms, data governance, genomic databases

## Abstract

The storage, sharing, and analysis of genomic data poses technical and logistical challenges that have precipitated the development of cloud-based computing platforms designed to facilitate collaboration and maximize the scientific utility of data. To understand cloud platforms’ policies and procedures and the implications for different stakeholder groups, in summer 2021, we reviewed publicly available documents (N = 94) sourced from platform websites, scientific literature, and lay media for five NIH-funded cloud platforms (the All of Us Research Hub, NHGRI AnVIL, NHLBI BioData Catalyst, NCI Genomic Data Commons, and the Kids First Data Resource Center) and a pre-existing data sharing mechanism, dbGaP. Platform policies were compared across seven categories of data governance: data submission, data ingestion, user authentication and authorization, data security, data access, auditing, and sanctions. Our analysis finds similarities across the platforms, including reliance on a formal data ingestion process, multiple tiers of data access with varying user authentication and/or authorization requirements, platform and user data security measures, and auditing for inappropriate data use. Platforms differ in how data tiers are organized, as well as the specifics of user authentication and authorization across access tiers. Our analysis maps elements of data governance across emerging NIH-funded cloud platforms and as such provides a key resource for stakeholders seeking to understand and utilize data access and analysis options across platforms and to surface aspects of governance that may require harmonization to achieve the desired interoperability.

## Teaser

Cloud-based data sharing and analysis platforms have been developed by the NIH to promote broad use of genomic and other biomedical data. We summarize policies and procedures of five such platforms, including those related to data submission, data ingestion, user authentication and authorization, data security, data access, auditing, and sanctions.

## Introduction

**Figure 1:**
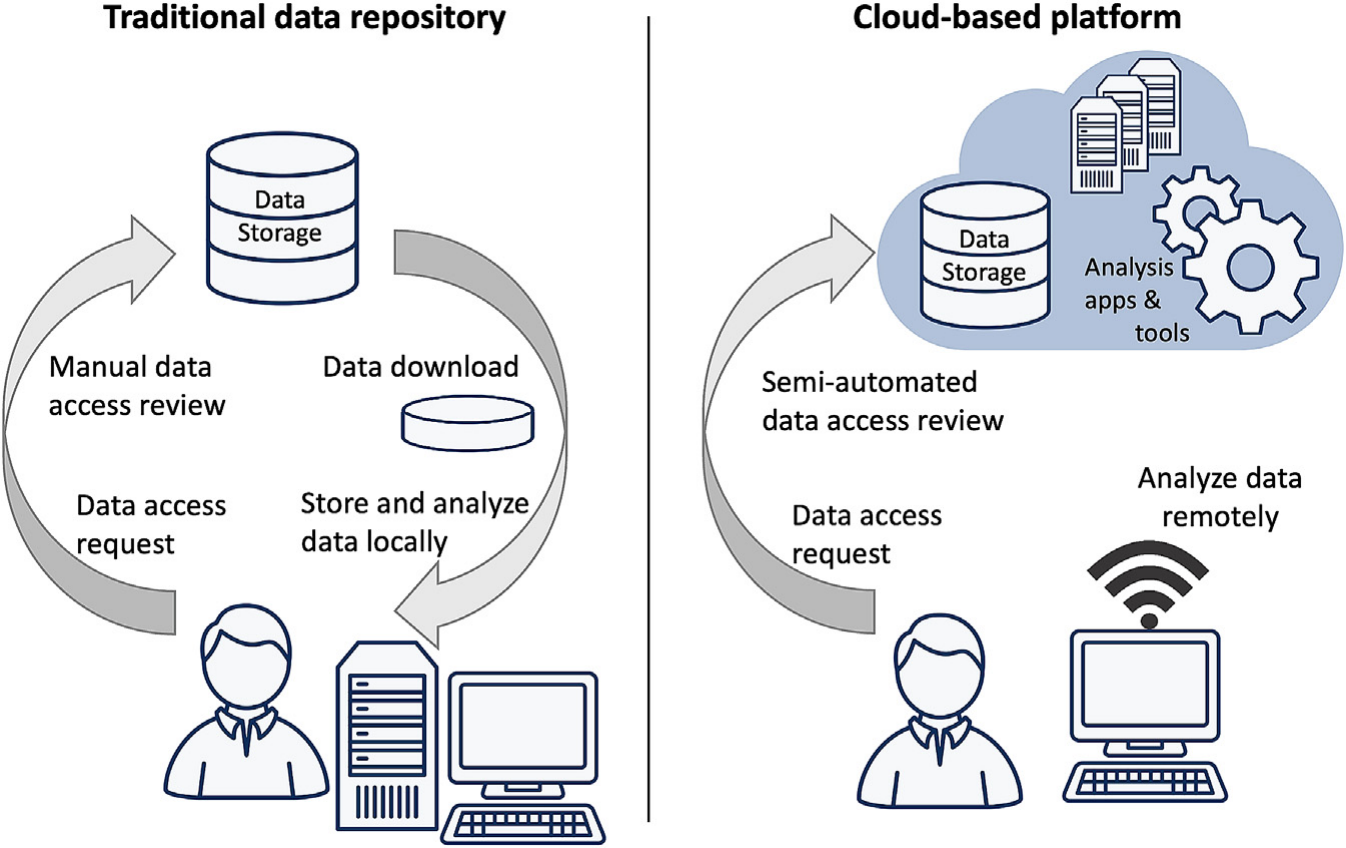
Traditional (left) versus cloud-based biomedical data sharing (right) In the traditional model, data are downloaded from a central repository and stored and analyzed locally. In the cloud-based model, data are stored and analyzed remotely in cloud environments.

Individual-level genomic, environmental, and linked phenotypic and health outcome data are being generated at an unprecedented pace and scale in human biomedical research. The storage, sharing, and analysis of such data poses profound technical and logistical challenges that have precipitated the development of new cloud-based computing and storage platforms designed to facilitate collaboration and maximize the scientific utility of costly-to-generate genomic and linked clinical data (Figure 1). Compared with pre-existing data sharing mechanisms such as the National Center for Biotechnology Information (NCBI) database of Genotypes and Phenotypes (dbGaP), emerging cloud-based platforms offer new and potentially more efficient alternatives for accessing, storing, and analyzing data, yet their specific policies and practices are not widely known, and the extent to which they adhere to previously proposed key functions of good genomic governance remains unexamined.^1^

We define a “cloud-based platform” as one that pairs cloud-based data storage with search and analysis functionality via cloud-based workspaces and portals. While individual components providing data access, storage, and analysis capabilities may be shared across different platforms, we identify a “platform” as a centralized system for data sharing associated with a specific NIH Institute or research initiative. (Notably, some of the entities we refer to as “platforms” may alternatively be described as “ecosystems,” in recognition of their multiple components.) At the time of analysis, there were five such platforms: the NIH Office of the Director’s All of Us Research Hub (AoURH), the National Human Genome Research Institute’s (NHGRI) Analysis Visualization and Informatics Lab-space (AnVIL), the National Heart, Lung, and Blood Institute’s (NHLBI) BioData Catalyst (BDC), the National Cancer Institute’s (NCI) Genomic Data Commons (GDC), and the NIH Common Fund Kids First Data Resource Center (Kids First DRC) (see Table 1).

**Table 1:** Platforms included in the document review (full name and abbreviation), primary funding body, and platform website (URL) at which document search was performed

Platform name	Platform abbreviation	Primary funder/NIH Institute	URL at which document search was performed
All of Us Research Hub	AoURH	NIH Office of the Director	https://www.researchallofus.org
Analysis Visualization and Informatics Lab-space	AnVIL	National Human Genome Research Institute (NHGRI)	https://anvilproject.org
BioData Catalyst	BDC	National Heart, Lung, and Blood Institute (NHLBI)	https://bdcatalyst.gitbook.io/biodata-catalyst-documentation
Kids First Data Resource Center	Kids First DRC	NIH Common Fund	https://kidsfirstdrc.org
Genomic Data Commons	GDC	National Cancer Institute (NCI)	https://gdc.cancer.gov
database of Genotypes and Phenotypes	dbGaP	National Center for Biotechnology Information (NCBI)	https://www.ncbi.nlm.nih.gov/books/NBK5295

Additional non-platform websites searched for documents (relevant peer reviewed and/or lay media) to analyze included PubMed, bioRxiv, medRxiv, Nature News, and Science News.

At least two key differences between cloud-based platforms and pre-existing data sharing mechanisms merit attention. First, cloud-based platforms “invert” data sharing in that users come to data stored in central cloud locations for analysis, rather than downloading data to store and analyze locally.^2^ Second, to expedite data access and analysis, streamlined mechanisms are being developed for both user authentication and authorization for use of data stored on such platforms.^3^^,^^4^ Borrowing some features from traditional models such as dbGaP but innovating others, cloud-based platforms therefore represent a partial continuation of prior genomic data sharing practices but also a sea change for data stewards in the novel ways that users can find, access, and analyze data.

A clear understanding of these platforms’ policies and practices is necessary to start unpacking the implications for many stakeholder groups, including research participants; researchers (data contributors and platform users); policymakers; funders; and researchers' institutions, which may be held accountable for data contribution and uses. It is also crucial as researchers and institutions begin to navigate the new NIH Data Management and Sharing Policy.^5^ The purpose of this paper is to (1) describe current data governance practices of emerging cloud-based platforms while (2) comparing these practices across new and pre-existing mechanisms to identify potential challenges and tradeoffs.

## Methods

This study used a cross-sectional qualitative directed content analysis of publicly available documents as they were available in June and July of 2021.^6^ The purpose of this analysis was to identify policies and practices of cloud-based genomic platforms in regard to data submission, data ingestion, user authentication and authorization, data security, data access, auditing, and sanctions.

### Platforms

We included five cloud-based platforms in our search that met our cloud platform definition above and were in development and/or early stages of active use at the time of our analysis: AoURH, AnVIL, BDC, GDC, and Kids First DRC. To situate these cloud-based platforms in the context of established data sharing mechanisms, we also included in our analysis dbGaP, whose data access request and review systems are also used by several cloud platforms. Initiatives that do not represent discrete platforms for data storage and analysis were excluded. We also did not include cloud-based platforms specific to a given academic research institution (e.g., the St Jude Cloud).^7^

### Document sampling

To find relevant documents, we searched the public-facing platform websites, as well as PubMed, preprint servers, and science news websites (see Table 1 for list and URLs). Search terms included widely recognized categories for data governance including: “data access,” “data use,” “data sharing,” “auditing,” and “permissions.” Documents were included if they relayed platform policies, procedures, or other governance-related topics, and they were excluded if they were summarized versions of longer documents, did not focus on the platform itself, or appeared to be primarily a form of marketing communication (e.g., press release) that promoted the platform’s achievements rather than described how it works. Our final document count was as follows: AoURH n = 17, AnVIL n = 12, BDC n = 21, Kids First DRC n = 7, GDC n = 20, and dbGaP n = 17. All documents so identified (N = 94) were downloaded between June and July 2021 and archived for consistency (for the full document breakdown, see Table S1). We recognize that platform documentation and policies are evolving, and therefore some of the information presented here may be incomplete and/or out of date. Please see the “limitations” section for more information.

### Analysis

Platform documents were coded and analyzed in ATLAS.ti^8^ using a codebook based on selected background literature.^1^^,^^9^^,^^10^^,^^11^ The codebook covered topics such as data protections, how data are made available and accessible, and platform history and organization. Some code examples include data access, roles and responsibilities, data ingestion, and auditing. Two coders (J.D. and S.N.) double-coded 12 of the same documents, two from each platform/mechanism, in order to assess inter-coder reliability and the robustness of the codebook (i.e., codes and definitions). After the initial pilot coding and subsequent minor adjustments to the codebook, J.D. coded the remaining documents with oversight from the rest of the research team.

## Results

Platform policies were compared across seven categories of data governance: data submission, data ingestion, user authentication and authorization, data security, data access, auditing, and sanctions. The results of these comparisons are described below and summarized in Table 3.

**Table 2:** Working definitions of key terms and concepts used in this paper, created from the authors’ understanding of the concepts as well as information provided in the documents analyzed

Term	Definition
Platform	A centralized system pairing cloud-based data storage with search and analysis functionality via cloud-based workspaces and portals. Associated with a specific NIH Institute or research initiative. May also be referred to as an “ecosystem.”
Data submission	The act of data generators providing their data to a platform.
Data ingestion	The action of obtaining, importing, transforming, cleaning, processing and otherwise curating submitted data to make it available on a platform.
Data harmonization	Ensures the compatibility of data from different submitters and/or studies via quality control, processing, and post-processing.
Data indexing	Assigning unique identifiers to data to support efficient discovery of said data.
Data curation	The process of receiving, transferring, organizing, integrating, removing, and preserving data residing within a platform.
Open tier	Open to anyone, without the need to register or authenticate your identity. Contain aggregate, de-identified data.
User authentication tier	Require users to log in with a username and password, usually via an external user account, but don’t include further requirements. Data varies from aggregate de-identified data to de-identified individual data.
User authorization tier	Require users to not only authenticate their identity but also be authorized to access the tier through a special request (such as a DAR).

**Table 3:** Summary table of document review results, with platforms as rows and columns as key features of data governance

Platform	Data submission	Data ingestion	User authentication and authorization	Data security	Data access	Auditing	Sanctions
AoURH	all data provided through the AoU Research Project	uses Observational Medical Outcomes Partnership Common Data Model to harmonize data; further cleans data to protect participant privacy	no login for public tier; eRA commons for registered tier; plans to change in the future eRA commons and more stringent access requirements than registered tier; users must be appropriately accredited and obtain separate authorization for controlled tier	user: no data screenshots, don’t publish or download ppt-level data, and don’t share logins platform: data is de-identified (though the platform notes that the term “de-identified” may not be fully accurate to describe its data); analysis of registered and/or controlled data only permitted on the platform	three access tiers: "public," "registered," and "controlled"; institutional AoU DUA (referred to as a “Data Use and Registration Agreement” or DURA) required; must complete research training (referred to as “Responsible Conduct of Research Training”), agree to DUCC, prove ID, share contact info and affiliations, and provide consent for release of this info; access authorization determined via "data passport" (user based); no DACs (project based)	reviews by RAB determine DUCC compliance; applicable corrective action is recommended; all user uploaded work will be logged and monitored; anyone, including researchers and the public, can ask RAB to review a study	termination of your account, public posting of your name and affiliation, user’s institution notified along with NIH or other federal agencies; financial or legal repercussions; other sanctions
AnVIL	must get approval from NHGRI and the AnVIL Ingestion Committee; data must conform to the NIH GDS Policy; participants must be explicitly consented for data sharing; the AnVIL Ingestion Committee assesses data; study must be registered in dbGaP	data ingestion committee evaluates applications and coordinates with dataset stewards (unspecified) to determine time frame for retention of data, long term storage, archival, and availability of data; uses Gen3 to ingest data	Google account for open tier; eRA commons for controlled tier; members of consortia are granted access directly by a designated consortium official	user: don’t re-identify ppts; platform: two-factor authentication, all data covered by Certificate of Confidentiality, systems are independently tested annually, system is continually tested and scanned, and is consistent with NIH Security Best Practices and GDS Policy	three access tiers: "open," "controlled," and "consortium"; three authorized user groups: developers, consortia, and external researchers; submit DAR and agree to DUC; DAC determines access via DAR, dbGaP consent codes, and DULs; piloting DUOS to streamline DAR approval; upload data in accordance with all national, tribal, state laws, and relevant institutional policies; consent groups placed into different workspaces	potential DMIs must be reported to DAC within 24 hours; Terra & Gen3 log access to data, go through audits, and are monitored for abnormal use; all activities are logged and regularly reviewed/monitored	access suspended or terminated and user’s institution notified
BDC	study must be registered in dbGaP	data "streamed in real time" or ingested in batches; BDC is a custodian of data, and it cannot control quality of data ingested; Data Management Core works with data providers to assess data with the intent of harmonizing; datasets are added by a user, ingested from a controlled source, or transferred from collaborative programs	eRA commons, Google, or ORCID for open tier; eRA commons for controlled tier	user: don’t re-identify ppts and don’t share logins; platform: Public Trust Clearance used for all staff and contractors, data within the cloud is encrypted, cannot download controlled access participant level data	two access tiers: "open" and "controlled"; submit DAR to dbGaP; Cloud Use Statement may be required; DAC determines access via DAR, dbGaP consent codes and DULs	all activities are logged and regularly reviewed/monitored	user institutions are accountable and may be subject to sanctions
GDC	accepts data from different cancer study groups; data submission adheres to the NIH and NCI GDS policies; aggregate data for patients aged 90+; submissions reviewed by considering a study’s size, quality, compatibility with data already hosted, and likely impact on the field; any investigator or consortium with cancer genomic data can apply for data submission; data submitters understand and agree that data will be made available to the scientific community; data submitters retain ownership of their data	submitted data is processed, validated, and harmonized before being hosted	no login for open tier; eRA Commons for controlled access	user: don’t re-identify ppts and comply with your DUA; platform: data is de-identified according to Health and Human services Safe Harbor guidelines, GDC does not house electronic health record and does not accept data for ppts over 90 years old	two access tiers: "open" and "controlled"; apply via dbGaP and DAC approves/denies; agree to DUA and NIH GDS policy and submit data sharing plan	GDC DMI standard operating procedure is referenced but details of auditing are not specified	data access is removed if data are discovered to contain PHI or PII or if data are shared out of compliance with sharing conditions set by DAC; sanctions for inappropriate data use not specified
Kids First DRC	DULs that impede the ability to access, use, or analyze data will not be prioritized; data consented only for disease-specific research; data that require a letter of collaboration or data that require local IRB approval will not be prioritized; projects that allow for broad data sharing will be prioritized	reports plans to use Human Phenotype Ontology and NCI Thesaurus for phenotype harmonization; lists a variety of workflows for genomic harmonization	no login for open tier; Google, ORCID, Facebook, or LinkedIn for KidsFirst tier; eRA commons for controlled tier	user: comply with NIH Security Best Practices for Controlled Access Data, report DMIs, don’t re-identify ppts, and don’t share logins; platform: N/A^a^	three access tiers: "open," "KidsFirst," and "controlled"; Submit DAR and DAC determines access via DAR, dbGaP consent codes, and DULs; agree to DUC	Gen3 & Cavatica monitor data use and ensure data access is appropriate; users instructed to report inadvertent data release or other DMI	N/A^a^
dbGaP	submitters required to certify they have considered the risks to individuals, their families, and populations associated with data submitted to dbGaP; all investigators receiving NIH support to conduct genomic research submit their de-identified study data to dbGaP; non-NIH-funded data can be submitted to dbGaP; requires the local IRB to certify consistency with laws and regulations	data undergo quality control and curation by dbGaP before being released to the public	no login for open tier; eRA Commons for controlled access	user: don’t re-identify ppts, create secure logins, don’t share logins, ensure data is secure and confidential, destroy locally stored data and officially close project when no longer needed, have a security plan and technical training, and policy controls in place before data migration, adhere to DAR for approved data use and to NIH security best practices, report DMIs, and users and user institutions accountable for ensuring data security, not the cloud service provider; platform: data is de-identified	two access tiers: "open" and "controlled"; submit DAR and agree to DUC and GDS policy; DAC determines access via DAR, dbGaP consent codes, and DULs	when notified, NIH reviews possible DMIs	user and institution are notified of problems, and appropriate steps are taken; users may face enforcement actions; access suspended or terminated

a N/A indicates information in the category was not found in the publicly available documents we reviewed in 2021. We recognize more information for these categories may be publicly available at the time of publication.

### Data submission

*Data submission* is the act of data generators providing their data to a platform (see Table 2 for summary definitions of italicized terms provided in italics in this section). Since 2008, all NIH-funded, high-throughput genomic studies have been required to submit their data to NIH-designated repositories such as dbGaP.^12^ Exceptions to this policy are made on a case-by-case basis, and non-NIH-funded studies are accepted into dbGaP at the discretion of NIH Institutes and Centers.^12^ Data submission must follow the sharing requirements and timelines in the NIH Genomic Data Sharing (GDS) policy.^13^

All platforms considered here require data generators to register their studies with dbGaP, with the exception of AoURH, which only contains data generated from the All of Us Research Program and does not accept external submissions. The usual dbGaP study registration process includes ethical oversight by the submitter’s Institutional Review Board (IRB) and dialog with an NIH Program Officer and a Genomic Program Administrator (GPA). Additional submission details from platform-specific documentation are as follows. Data generators wishing to deposit data within AnVIL must get approval from the AnVIL Ingestion Committee. That Committee assesses whether the data are a good fit; this is determined in part by the amount of data, ethical oversight during data collection, how participants were consented, and what data use limitations (DULs) are included.^14^ BDC documentation highlights that when submitting data, data generators must work with an NHLBI GPA and register their data with dbGaP, the “central registration authority” for BDC.^15^ The GDC accepts data from genomic cancer studies; priority is given to new data types, and the study’s size, quality, and ability to further understanding of cancer are taken into account.^16^ The Kids First DRC notes that “projects that allow for the broadest leveling of sharing … will be prioritized for Kids First Support” and states that restrictions like disease-specific consent or requiring a letter of collaboration “impede the ability for the Kids First program to accomplish its goals.”^17^

### Data ingestion

Once data are submitted and accepted, they go through an intake or *data ingestion* process before becoming available to researchers. While not defined explicitly or consistently across platforms, data ingestion generally entails transforming, cleaning, processing, harmonizing, indexing, and/or otherwise curating data submitted by data generators in order to make it accessible on a platform. Platforms typically require data to undergo quality control and harmonization as part of ingestion. *Data harmonization* ensures that data from different studies and generators are compatible. Data are also *indexed* as part of the ingestion process, which involves assigning unique identifiers to each data file.^18^ AoURH uses the Observational Medical Outcomes Partnership Common Data Model to harmonize data before taking further steps to “ensure participant privacy is protected.”^19^ AnVIL’s Data Ingestion Committee, which includes AnVIL team members and NHGRI program officers, evaluates applications for ingestion as a form of quality control.^20^ BDC, while recognizing that their data does go through quality control, states that they are data “custodians” and “cannot control the quality of data ingested.”^21^ BDC contains data ingested from dbGaP or directly from participating consortia, and it has plans for a separate center, the Data Management Core, that will help researchers with ingestion requirements.^15^ The GDC, while not addressing quality control explicitly, notes that it can take up to 6 months after processing data before it is released to researchers.^22^ Kids First DRC reports plans to use the Human Phenotype Ontology and NCI Thesaurus for phenotype harmonization, and it lists a variety of workflows that can be used for genomic harmonization.^17^

### User authentication and authorization

All platforms divide ingested data into two or three data tiers; however, the types of data contained in those tiers and requirements to access vary between platforms. *Open tiers* are open to anyone, without the need to register or otherwise authenticate the identity of the data user. *User authentication tiers* require users to log in with a username and password, usually via an external user account, but they do not include further requirements or subsequent identity verification. *User authorization tiers* require users to not only authenticate their identity but also to be authorized to access the tier, usually through a specific request (such as a data access request [DAR]). User authorization tiers are included in every platform; platforms vary with regard to their use of open and/or user authentication tiers.

### Open tiers

AoURH, GDC, Kids First DRC, and dbGaP have open tiers. These open tiers allow users to browse the studies contained in these tiers without logging in or making any special requests for data access or use. AoURH’s open tier allows users to view “summary statistics and aggregate information that poses negligible risks to the privacy of research participants.”^23^ With the GDC and Kids First DRC open tiers, users can search for studies or use open datasets.^24^^,^^25^^,^^26^

### User authentication tiers

AnVIL, BDC, and Kids First DRC have user authentication tiers. Data contained in these tiers vary from aggregate de-identified data to de-identified individual data. AnVIL and BDC, which primarily provide aggregate de-identified data or unrestricted individual-level data to this tier (e.g., 1000 Genomes Project data in AnVIL), each require authentication with Google, ORCID, or eRA Commons credentials (see Table 3).^15^^,^^27^^,^^28^^,^^29^^,^^30^ The Kids First DRC has a “KidsFirst” tier that allows users to “search de-identified data” and can be accessed by logging in using Google, ORCID, Facebook, or LinkedIn credentials.^24^^,^^31^ This is the only platform in our analysis that allows user authentication using Facebook or LinkedIn credentials.

### User authorization tiers

All platforms have user authorization tiers. User authorization tiers require that users both authenticate their identity and submit a request to access data for a specific research use, such as a DAR (see data access below for more detail). In all cases, access to individual de-identified data is allowed upon authorization.

The AoURH differs from other platforms in having two different user authorization tiers that, at the time of this analysis, both require an eRA Commons account. First is the “Registered” tier, which contains individual-level data and requires an eRA Commons account and a request to access the data.^23^^,^^32^ AoURH also has a second “Controlled” tier that contains individual-level genetic data, which according to AoURH, requires more “stringent” user authentication than the individual-level data in their “Registered” tier.^32^ At the time of review, to access this “Controlled” tier, users must log in with an eRA Commons account as well as be “appropriately accredited” and obtain “additional approval.”^23^ What this extra accreditation and approval might entail was unclear from the documentation available to our review. We consider both of these tiers to be authorization rather than authentication tiers because of the required project description to initiate a workspace in either tier, meaning that access to data is tied to a specific proposed use analogous to a dbGaP Research Use Statement.

Another variation is AnVIL’s “Consortium” tier that is only open to members of specific research consortia who have placed their data on the platform, typically ahead of release to the scientific community. Consortia members gain access directly from a consortium official, and it is the responsibility of the consortium to manage who is allowed access to these data.^33^

### Data security

Platform policies reference data security in two distinct ways: individual users’ responsibilities with respect to data security and platform responsibilities designed to ensure a secure environment. All platforms encourage a standard set of data security practices that users are responsible to uphold, such as not sharing login information, not taking screenshots of data, and not attempting to re-identify participants.

Platform responsibilities for ensuring data security can vary, but many aspects are similar across the board due to federal regulations in the United States. For example, AoURH, AnVIL, BDC, GDC, and dbGaP reference following the Federal Information Security Modernization Act (FISMA) or National Institute of Standards and Technology (NIST) guidelines. FISMA has set rules around information security, and NIST outlines guidance for complying with those rules. Other similar platform data security measures include encrypting data, performing routine system security checks, and de-identifying data in a manner consistent with the GDS policy.

Some platforms have clear rules around data download. For instance, AoURH and BDC specify that they do not allow data in user authorization tiers to be downloaded at all.^32^^,^^34^ However, BDC also notes that it is technically possible for users to download platform data, and therefore the responsibility lies with the user to not download the data.^34^ In contrast to the cloud-based platforms, in dbGaP the data are typically meant to be downloaded onto the applicant’s local system. For this reason, dbGaP makes clear that users and their institutions are responsible for their data’s security. Users are advised to avoid putting controlled access data on portable devices, and they are encouraged to have an institutional data security plan in place before migrating data.^35^ dbGaP also requires that projects be closed out officially when data are no longer needed and that all locally stored files are destroyed.^35^ It was unclear how data access on cloud-based platforms would cease at the conclusion of a project; we found no equivalent information about project close-out procedures specific to cloud-based platforms.

A range of other data security precautions from the platform side were noted; for example, GDC does not accept data from participants over the age of 90, and similarly AoURH limits what can be reported on participants aged 90+.^22^^,^^32^ BDC specifies that they use network firewalls and require all platform staff and contracts to have public trust clearance, which includes a thorough background check.^15^ All platforms validate user identity, at least in principle, and post descriptions of research projects publicly.

### Data access

All platforms contain user authorization tiers that include individual-level data, such as germline genomic data and certain phenotype or clinical data, depending on the study and/or the platform. To access these data, most platforms require the user to submit a DAR to dbGaP. Typically, a DAR is first reviewed by a signing official from the applicant’s institution; alternatives to this model are discussed below. A data access committee (DAC) then reviews the DAR and determines whether or not to approve it based on the DULs for the requested dataset.^36^ Which DAC reviews a DAR typically depends on which NIH Institute a study is registered with; Kids First DRC is distinct in having a dedicated Kids First DRC DAC.^17^ Once approved, users must also agree to adhere to the NIH GDS policy, and a data use agreement (DUA), depending on the platform. AoURH requires that the user’s *institution* enters into a DUA with the All of Us Research Project (AoURP), referred to as a “Data Use and Registration Agreement” or DURA. Other platforms refer to DUAs in different contexts: BDC stipulates that users must ensure that DUAs are “approved and maintained,”^37^ GDC notes that applying for access via dbGaP requires users to sign a DUA established by data owners,^38^ and the AnVIL states that DUAs are required “as necessary.”^33^ Kids First DRC did not provide information on the need for DUAs. AoURH also requires that researchers adhere to the All of Us Data User Code of Conduct, which prohibits data users from “attempting to re-identify participants or their relatives'' and encourages them to “be careful when distributing the results of their work” to “prevent others from using this information to re-identify All of Us participants''.^39^

There are two main variations from the process noted above: (1) models where data *users*, instead of data *uses*, are authorized and (2) models that have automated or semi-automated data access review. In the first category are AoURH and the AnVIL. The AoURH does not use the dbGaP DAC system and instead uses a “data passport” model that grants access to vetted “Authorized Users” rather than granting data access on an individual project basis.^32^^,^^40^ This data passport system is possible due to the single broad consent for data access and use that governs all data on the AoURH platform; other platforms do not currently have this ability. To become an “authorized user,” the user’s institution must have a DURA with the AoURP. With this in place, users must then do the following before becoming “Authorized Users”: establish their identity (using eRA Commons to validate), consent to public display of their name and description of their research projects, consent to public release of their name in case of a DUCC violation, complete the All of Us Responsible Conduct of Research Training, and sign an agreement testifying they have done what is required.^23^^,^^32^ After this is complete, “Authorized Users” will be able to access AoURH’s “Registered” and “Controlled” tiers, create workspaces on the AoURH, and carry out research with the data.^23^^,^^32^ A similar variation is AnVIL’s in-development “library card” concept, which like the data passport, would allow researchers to be pre-authorized to request user authorization tier data.^2^ This process uses the Global Alliance for Genomics and Health (GA4GH) Passport Visa system and aims to reduce the number of steps researchers have to go through before gaining access to data, while still ensuring they are permissioned to do so.^2^^,^^41^ However, unlike the AoURH passport approach, pre-authorized AnVIL users are still required to complete a dbGaP DAR.^2^^,^^20^

The second variation from the process above is the Data Use Oversight System (DUOS), which is being piloted by the AnVIL. DUOS uses the GA4GH Data Use Ontology (DUO) algorithm to compare DARs with the requested dataset’s consent codes and DULs. The algorithm can then suggest that the DAC either approve or deny that DAR, with the hope that this expedites data access review and reduces the burden on DACs.^2^

### Auditing

All platform actions are regularly monitored and audited for abnormal use and to ensure researchers are only accessing the data for which they have appropriate permissions. The AnVIL and dbGaP specifically ask that all data management incidents (DMIs), such as unauthorized data sharing or data security breaches, be reported as they are found, and therefore these platforms rely partially on research teams and their institutions to help with auditing.^42^^,^^43^ Reported DMIs are reviewed by the affected project’s DAC, and corrective action is determined by the DAC if necessary. The GDC mentions that if any data are found to contain protected health information (PHI) or personally identifiable information (PII), that data will be removed and reported via the GDC DMI procedure (which includes notifying submitters and correcting the issue before re-release).^22^ They do not specify how this kind of DMI might be identified in the first place.

The AoURH is once again a unique platform when it comes to auditing. In addition to performing regular platform audits, their Resource Access Board (RAB) conducts *ad hoc* reviews of workspaces and research project descriptions to identify any that may be in violation of their DUCC. If the RAB finds a project is noncompliant, they can recommend corrective action. This model is especially unique as anyone, including research participants, the public, or researchers themselves can request to have any project reviewed by the RAB.^32^^,^^44^

### Sanctions

Neither the GDC nor the Kids First DRC described sanctions for misuse in the public-facing documents included in our search. However, the remaining platforms agree on a range of such sanctions, including public posting of the sanctioned user’s name and affiliation, suspension or removal from platform use, and notifying the user’s home research institution. AoURH implies that NIH and other federal agencies may be notified as well and that “financial or legal repercussions” may ensue.^23^ In addition, AoURH reserves the right to pursue “other sanctions” as they see fit.^23^ The BDC notes that user institutions are accountable in addition to users and therefore may face sanctions as well.^45^

## Discussion

Major public investment in cloud-based platforms is enabling the storage, access, and analysis of large amounts of individual-level genomic, environmental, and linked phenotypic data. Currently several such platforms are in development or early use, and our analysis focused on the publicly accessible documentation associated with the five most prominent: the AoURH, the AnVIL, BDC, the GDC, and the Kids First DRC. The aim was to describe the heterogeneous landscape of these new cloud-based data sharing mechanisms with the intent of understanding currently operating policies and procedures enacting data governance and how these may differ from pre-existing mechanisms.

Our analysis suggests many similarities across the platforms, including reliance on a formal data ingestion process, multiple tiers of data access that often require some degree of user authentication and/or authorization, platform and user data security measures, and auditing for inappropriate data use. Many of the platforms use eRA Commons credentials for user authentication and rely on dbGaP for study registration and the adjudication of DARs, so as to authorize use of controlled access data. Platforms differ in the way in which they choose to organize their tiers of data, as well as the specifics of user authentication and authorization for different types of data. The AoURH, unlike other platforms, does not use dbGaP to manage data access, choosing to rely instead on an investigator-centered “data passport” model and post-hoc vetting of publicly described research projects for compliance with its user code of conduct. This novel data access mechanism is enabled by use of a common, broad consent agreement, versus other platforms that generally provide access to numerous studies with varying (and often legacy) consent. What explains other differences between the platforms is not always immediately obvious. Regardless, the complexity and heterogeneity we observe within and across platforms could ultimately limit the goal of efficiently combining and analyzing large enough datasets to advance precision medicine goals.

The siloed nature of current NIH-supported cloud-based data sharing mechanisms is well recognized, and an effort is underway to identify avenues to enhance interoperability, i.e., the NIH Cloud Platform Interoperability (NCPI) effort.^46^ Platforms involved with this effort include the AnVIL, BDC, the NCI Cancer Research Data Commons (which includes the GDC), Kids First DRC, and NCBI (which manages dbGaP). The NCPI aims to establish a “federated data ecosystem” by integrating aspects of cloud platforms such as user authentication, data discovery, datasets, and workflows using different application programming interfaces (APIs).^46^ Using these APIs, researchers can access data on one platform and analyze it with the tools of another platform without having to download or host the data externally. Pilot innovations designed to streamline data access, which might ultimately be shared across platforms, include the NIH Research Authorization System (RAS), a simplified approach to user authentication,^2^^,^^15^^,^^47^ as well as the AnVIL library card concept, which like the AoURH data passport model would pre-authorize investigators for controlled data access.^2^^,^^4^ Similarly, the AnVIL DUOS system of semi-automating data access review could ultimately expedite DAC review for other platforms that utilize dbGaP for DARs,^2^^,^^3^ which will remain essential for federated data accrued under varying consent understandings and thus subject to different DULs. Notably, ease of access was identified as a driving feature in the selection of genetic databases in a recent empirical study of genetic researchers.^48^ Whether or how platforms such as the AoURH, which employ fundamentally different approaches to data access review, can participate in cross-platform data or analytic pipeline sharing remains an open question. Achieving interoperability will require both policy and technical solutions and will likely surface tensions between enhancing security and promoting participant trust in a platform versus maximizing the scientific utility of a resource.

In a recent commentary addressing better governance of human genomic data, O’Doherty et al. (2021) note that enhanced data sharing raises concerns about potential risks, including privacy violations, misuse of data, and unauthorized data access.^1^ They describe five “key functions of good governance” that governance frameworks, ideally, would fulfill, including (1) enabling data access, (2) compliance with applicable national laws and international agreements, (3) supporting appropriate data use and mitigating possible harms, (4) promoting equity in access to, and use and analysis of, data, and (5) using data for public benefit. They also cite transparency as a “meta-function of good governance” and one in which “unlike the other functions, cannot legitimately vary by context or be balanced against other dimensions of good governance.” The cloud-based platforms in our analysis are each designed to enable facile data access (function 1), particularly of very large genomic data, but they vary in the degree to which their policies and procedures are transparently described. Indeed, a primary motivation for our analysis was to promote greater transparency about these new data sharing mechanisms by conducting a comprehensive (and comparative) assessment of the public documentation provided by these platforms. Interestingly, but perhaps unsurprisingly, the greatest transparency we observed was for a category of platform information that we did not originally set out to measure, i.e., cost. Most platforms explicitly outlined the ways that users must pay for cloud storage, data egress (or download, where allowed), and computing time. Platforms devote substantial documentation to how these costs work, how to set up billing accounts with Cloud Service Providers, and what the user’s responsibilities are in this respect. Platforms also generally described the use of the “cloud credits” they offered to new users to help reduce initial barriers to using these platforms.

Several of the other key functions that O’Doherty et al. (2021) describe are also well-represented across the platforms whose documentation we reviewed.^1^ All of the platforms we examined, for example, are sponsored by the NIH and so subject to US federal data sharing requirements and data security standards (function 2). Specifically, most platforms explicitly noted following the FISMA standards and/or NIST guidelines. Similarly, in making genomic and linked phenotypic data available for analysis in the cloud, these efforts also—at least in theory—promote equity in access to, and use and analysis of, shared data (function 4). Compared with earlier data sharing mechanisms, which required investigators to have access to local data storage and computational resources sufficient to manage and analyze very large datasets, each of these cloud-based platforms provide much easier (albeit remote) access to data as well as access to a wide variety of analytical tools and pipelines. Although computation time must still be paid for, the costs of data storage are typically borne by the platform, and both junior investigators and researchers at institutions without the necessary infrastructure can now access and analyze data that were previously out of reach. Lack of publicly accessible data about current or anticipated users makes it difficult to determine if this promise of more equitable data access has yet been achieved. Notably, initiatives such as the Genomic Data Science Community Network are working toward these ends.^49^

O’Doherty et al. also note that good governance frameworks would ideally “clarify how its operations enhance public trustworthiness and the public good” and, hence, enhance public benefit (function 5).^1^ While all the platforms we reviewed have user authentication and auditing mechanisms designed to promote public confidence in the security of the data they house and share, we encountered relatively little information about the degree to which these platforms solicit participant and/or public input on their operations. The AoURH RAB, which audits research projects to ensure compliance with AoU policies, does include research participant representatives.^44^ Where we saw the greatest heterogeneity in platform functionality was in supporting appropriate data use and mitigating possible harms (function 3), with different approaches taken to user authentication and data access review. User authentication for most data access required use of a vetted user credential, such as from the eRA Commons, but some platforms also accepted Google, Facebook, or LinkedIn credentials, at least for non-controlled access data (typically aggregated and de-identified). While widening access to non-academic researchers, this may also risk exposing data to users whose identities cannot ultimately be verified. Similarly, while the goal of streamlining data access by authorizing users rather than specific research uses (as adopted by the AoURH) works well in that research setting, it does leave open the possibility that stigmatizing or harmful research could nevertheless be pursued. While that potential is also there with DAC-vetted DARs, a prospective review does provide the opportunity to give feedback to investigators who may not otherwise recognize the potential for harm. The AoURH Responsible Conduct of Research Training, required for Registered and Controlled tier users, may similarly “front load” protective measures against data misuse, but again, it is not feedback specific to proposed projects. In either case, not enough information about platform auditing procedures was available to judge the extent to which harmful research uses could be detected and mitigated or who might be held responsible if something goes wrong.

### Limitations

This analysis was not without limitations. Mainly, our methods of sourcing documents may have led to some relevant information being overlooked. We were limited to publicly available documentation, and in an effort to keep the scope of this analysis manageable, we primarily sourced documents from platform websites (supplemented by academic literature and limited science news websites). We recognize that platform documentation is evolving as policies are established and as platforms continue to develop; therefore, our current analysis may omit more recently added or updated information. As a result, some of our outstanding questions may have answers available in sources we did not search or in sources we did search but that have since been updated. It is also possible our understanding gleaned from available sources is incomplete or not wholly accurate. However, we contend that what we have inferred from available documentation is comparable to what other researchers and platforms users may understand.

In addition, we learned a lot about genomic cloud computing platforms during this analysis that would alter our approach were we starting anew. As part of learning more about these platforms, we learned that there may be other sites we should have incorporated into our document search (e.g., the NCPI). In addition, our analysis does not include more recently developed cloud platforms that would have otherwise fit our scope criteria (e.g., NIH INCLUDE).^50^ We are also aware there is likely a wealth of information about these platforms available from non-public sources.

### Future directions

Our analysis maps elements of data governance across emerging NIH-funded cloud platforms and as such provides a key resource for a range of future investigations and stakeholders. We aim to enable investigators seeking to understand and utilize data access and analysis options across platforms. For policymakers, we surface governance decisions that may require harmonization within and across platforms to achieve the desired interoperability. To supplement and extend what we learned from our analysis of publicly available documentation reported here, we are conducting additional research, including key informant interviews with platform developers, users, and other stakeholders, to gain deeper and first-hand understanding of platform design and use. It would also be worthwhile for future work to look at costs of these cloud platforms versus traditional platforms, as this is something we did not incorporate into the analysis. Equipped with more complete information about the governance of these new data sharing mechanisms, we will be well-positioned to contribute to ongoing interoperability efforts and help promote broad public support for such initiatives.

## Data Availability

The dataset and codes supporting the current study are available from the corresponding authors on request.

## Supplemental information

Table S1. Documents included in the document review, with associated metadata
